# Амиодарон-индуцированный тиреотоксикоз 2 типа: ретроспективный анализ эффективности терапии глюкокортикоидами

**DOI:** 10.14341/probl13267

**Published:** 2024-01-24

**Authors:** А. С. Ермолаева, В. В. Фадеев

**Affiliations:** Первый Московский государственный медицинский университет им. И.М. Сеченова (Сеченовский Университет); Первый Московский государственный медицинский университет им. И.М. Сеченова (Сеченовский Университет)

**Keywords:** амиодарон, щитовидная железа, амиодарон-индуцированный тиреотоксикоз 2 типа, глюкокортикоиды

## Abstract

**ОБОСНОВАНИЕ:**

ОБОСНОВАНИЕ. Амиодарон-индуцированный тиреотоксикоз 2 типа остается серьезной проблемой эндокринологии и кардиологии. В связи с увеличением продолжительности жизни населения увеличивается распространенность нарушений ритма сердца, по поводу которых назначается амиодарон. Развитие тиреотоксикоза усугубляет имеющуюся у пациентов сердечно-сосудистую патологию: приводит к прогрессированию дисфункции левого желудочка, рецидивам нарушений ритма, увеличивая риск неблагоприятных исходов. Тактика дальнейшего ведения пациентов сложна: необходимо решить вопрос об отмене либо продолжении приема антиаритмика, необходимого пациенту с нарушением ритма сердца в анамнезе, а также грамотной терапии возникшей патологии щитовидной железы. Пероральные глюкокортикоиды являются препаратами первой линии для лечения пациентов с умеренным и тяжелым течением амиодарон-индуцированного тиреотоксикоза 2 типа. Несмотря на появление клинических рекомендаций, мнения по поводу тактики ведения пациентов разнятся как среди кардиологов, так и среди эндокринологов. Зачастую пациентам одновременно с глюкокортикоидами назначаются тиреостатические препараты, хотя это назначение не имеет патогенетических оснований.

**ЦЕЛЬ:**

ЦЕЛЬ. Оценить эффективность различных вариантов терапии у пациентов с амиодарон-индуцированным тиреотоксикозом 2 типа.

**МАТЕРИАЛЫ И МЕТОДЫ:**

МАТЕРИАЛЫ И МЕТОДЫ. В ретроспективное исследование включены 38 пациентов (20 мужчин и 18 женщин в возрасте от 35 до 85 лет) с амиодарон-индуцированным тиреотоксикозом 2 типа. Всем пациентам проводились анализ анамнестических, антропометрических данных, комплексная лабораторно-инструментальная диагностика. По вариантам терапии ретроспективно сформированы 3 группы: без терапии (n=19), получавшие глюкокортикоиды (n=11) и комбинацию глюкокортикоидов и тиреостатиков (n=8). Срок наблюдения составил 6–18 мес, включая период лечения. Эффективность лечения в группах оценивалась по времени достижения эутиреоза на фоне терапии глюкокортикоидами и длительности тиреотоксикоза; проводился поиск потенциальных предикторов отсроченного ответа на терапию глюкокортикоидами и длительного течения тиреотоксикоза.

**РЕЗУЛЬТАТЫ:**

РЕЗУЛЬТАТЫ. Средний возраст составил 62,0 [52,9; 66,3] года. Достоверное снижение уровня свободного тироксина наблюдалось через 1 мес от начала терапии в обеих группах: с 38,1 [32,1; 58,4] до 23,4 [19,6; 29,3] пмоль/л (р<0,001) в группе, получавшей глюкокортикоиды; с 73,9 [42,2; 75,6] до 39,3 [22,4; 47,2] пмоль/л (р<0,001) в группе комбинированной терапии. Время достижения эутиреоза было большим в группе комбинированной терапии (р=0,047), не зависело от дозы (р=0,338) и длительности приема тиамазола (р=0,911), отсроченность ответа на терапию коррелировала с возрастом (ρ=-0,857; p=0,007) и временным интервалом от возникновения клинической симптоматики тиреотоксикоза до назначения глюкокортикоидов (ρ=0,881; p<0,001).

**ЗАКЛЮЧЕНИЕ:**

ЗАКЛЮЧЕНИЕ. Полученные результаты демонстрируют зависимость терапевтического ответа на глюкокортикоиды от возраста пациента и времени их назначения относительно длительности тиреотоксикоза, нецелесообразность дополнительного применения тиреостатических препаратов при амиодарон-индуцированном тиреотоксикозе 2 типа.

## Abstract (en)

**BACKGROUND:**

BACKGROUND: Type 2 amiodarone-induced thyrotoxicosis remains a significant problem of endocrinology and cardiology. Due to the increase a life expectancy of the population, the prevalence of cardiac arrhythmias and prescribing of amiodarone are increasing. Thyrotoxicosis aggravates the existing cardiovascular disease in patients, leads to the progression of left ventricular dysfunction, relapses of arrhythmias, increasing the risk of adverse outcomes. The tactic of further management of patients is complicated: it is necessary to resolve the issue of canceling or continuing the use of antiarrhythmic drugs necessary for a patient with a history of cardiac arrhythmia, as well as competent therapy of the thyroid pathology that has arisen. Oral glucocorticoids are the first-line drugs for the treatment of patients with moderate and severe type 2  amiodarone-induced thyrotoxicosis. Despite the appearance of clinical recommendations, opinions on the management of patients are differ, both among cardiologists and among endocrinologists. Often thyrostatics are prescribed to patients simultaneously with glucocorticoids, although it doesn’t have pathogenetic basis.

**AIM:**

AIM: To evaluate the efficacy of various therapy options in patients with type 2 amiodarone-induced thyrotoxicosis.

**MATERIALS AND METHODS:**

MATERIALS AND METHODS: The retrospective study included 38 patients (20 men and 18 women aged 35 to 85 years) with type 2 amiodarone-induced thyrotoxicosis. All patients underwent an analysis of anamnestic, anthropometric data, complex laboratory and instrumental diagnostics. According to the treatment options, 3 groups were retrospectively formed: without therapy (n=19), taking glucocorticoids (n=11) and combination of glucocorticoids and thyrostatics (n=8). The follow-up period was 6–18 months, including the treatment. The efficacy of treatment in the groups was evaluated by the time of reaching euthyroidism on the background of glucocorticoid therapy and duration of thyrotoxicosis; the search was conducted for potential predictors of delayed response to glucocorticoid therapy and long-term course of thyrotoxicosis.

**RESULTS:**

RESULTS: The average age was 62.0 [52.9; 66.3] years. The level of free thyroxine was significantly decreased after 1 month from the start of therapy in both groups: from 38.1 [32.1; 58.4] to 23.4 [19.6; 29.3] pmol/l (p<0.001) in the group taking glucocorticoids; from 73.9 [42.2; 75.6] to 39.3 [22.4; 47.2] pmol/l (p<0.001) in the combination therapy group. The time of reaching euthyroidism was longer in the combination therapy group (p=0.047), didn’t depend on the dose (p=0.338) and duration of taking thiamazole (p=0.911), the delayed response to therapy correlated with age (p=-0.857; p=0.007) and time interval from the appearance of clinical symptoms of thyrotoxicosis to the start of glucocorticoid therapy (p=0.881; p<0.001).

**CONCLUSION:**

CONCLUSION: The results demonstrate the dependence of glucocorticoid response on the age of the patient and start time of therapy relative to the duration of thyrotoxicosis, inexpediency of additional prescribing thyrostatics in type 2 amiodarone-induced thyrotoxicosis.

## ОБОСНОВАНИЕ

Нарушения ритма сердца — одна из причин смертности и инвалидизации пациентов. Фибрилляция предсердий является наиболее распространенной аритмией, встречающейся в клинической практике, связана с риском развития неблагоприятных сердечно-сосудистых исходов, включая инсульт, инфаркт миокарда, сердечную недостаточность, независимо ассоциирована с увеличением риска смертности в 3,5 раза. В связи с увеличением продолжительности жизни населения ожидается дальнейший рост распространенности нарушений сердечного ритма в возрасте старше 65 лет, что оказывает ощутимую нагрузку на пациентов, общественное здоровье и экономику здравоохранения. В связи с чем требуется многогранный, целостный и междисциплинарный подход к ведению данной категории пациентов [1–3].

Амиодарон — широко применяемый эффективный антиаритмик при предсердных и желудочковых нарушениях ритма, в том числе и у пациентов со структурным поражением сердца, включая тяжелую хроническую сердечную недостаточность с низкой фракцией выброса левого желудочка (ФВЛЖ), а также с имплантируемыми медицинскими изделиями для проведения сердечной ресинхронизирующей терапии (электрокардиостимулятор, кардиовертер-дефибриллятор) [1][2][4][5]. Он представляет собой йодированное (75 мг йода в 200 мг препарата) жирорастворимое производное бензофурана. Фармакология амиодарона уникальна: сочетает свойства 4 классов антиаритмических препаратов (блокирование калиевых, натриевых каналов, неконкурентное β-адрено­блокирующее действие, подавление медленных кальциевых каналов и α-блокирующий эффект). Он обладает высокой амфифильностью и большим объемом распределения (формирование внутриклеточных депо с медленным высвобождением), длительным периодом полувыведения (приблизительно 50–100 дней), однако в связи со структурной схожестью с тиреоидными гормонами влияет на их синтез и метаболизм, может оказывать прямой цитотоксический эффект, вызывая апоптоз/некроз клеток [6–8].

Амиодарон-индуцированный тиреотоксикоз (АмИТ) — нередкое осложнение терапии препаратом. Выделяют два возможных патогенетических варианта АмИТ. При первом типе (АмИТ1) тиреотоксикоз обусловлен чрезмерным синтезом гормонов (гипертиреоз) в ответ на йодную нагрузку на фоне латентно предсуществующей патологии щитовидной железы (функциональная автономия, болезнь Грейвса), при втором типе (АмИТ2) — высвобождением «запасов» тиреоидных гормонов в кровоток в результате деструкции тиреоцитов [9][10]. Частота возникновения АмИТ2 варьирует от 0,6 до 21% [11], это преобладающий вариант нарушения функции щитовидной железы [12][13].

Специфические предикторы развития АмИТ2 неизвестны [14]. Временной интервал от начала приема антиаритмика до развития тиреотоксикоза варьирует от нескольких месяцев до нескольких лет. Опасность АмИТ2 заключается в усугублении проявлений сердечной недостаточности, декомпенсации аритмий, увеличении смертности, особенно среди пожилых пациентов [15]. Определение типа АмИТ нередко является диагностической дилеммой для клиницистов. Не существует «золотого стандарта» для верификации диагноза, но именно от нее зависит терапевтическая тактика. Для АмИТ2, как правило, характерны отсутствие предшествовавшей патологии щитовидной железы, низкий уровень антител к тиреоидной пероксидазе (АТ-ТПО), тиреоглобулину, рецептору тиреотропного гормона (АТ-рТТГ), отсутствие гиперваскуляризации по результатам доплеровской сонографии, низкий захват пертехнетата (99mTcO4) или технетрила (99mTc-sestaMIBI) по данным сцинтиграфии [16–18].

Продолжение терапии амиодароном, несмотря на развившийся АмИТ2, обсуждается в каждом конкретном случае совместно с кардиологом, поскольку дальнейший прием антиаритмика не препятствует восстановлению эутиреоза [19][20], но может отсрочить его наступление [21].

Препаратами первой линии для лечения пациентов с АмИТ2 являются глюкокортикоиды (ГК). Несмотря на появление клинических рекомендаций, мнения по поводу тактики ведения пациентов (показания к назначению ГК, стартовая доза, продолжительность терапии и алгоритм отмены) разнятся как среди кардиологов, так и среди эндокринологов. Неуверенность в диагнозе АмИТ2 и недостаточный опыт зачастую приводят к одновременному назначению ГК и тиреостатиков, хотя гиперфункция щитовидной железы при этом заболевании отсутствует. У пациентов с тяжелой кардиальной патологией быстрое восстановление эутиреоза имеет первостепенное значение, но не всегда достижимо с помощью медикаментозной терапии. Факторы, позволяющие спрогнозировать эффективность и длительность терапии ГК, могут иметь решающее значение для выбора оптимальной тактики ведения пациента [9][10][20][22].

## ЦЕЛЬ ИССЛЕДОВАНИЯ

Оценить эффективность терапии ГК у пациентов с АмИТ2 в виде монотерапии и в комбинации с тиреостатиками на основании ретроспективного исследования.

## МАТЕРИАЛЫ И МЕТОДЫ

## Место и время проведения исследования

Место проведения

Исследование было проведено на базе клиники эндокринологии ФГАОУ ВО «Первый МГМУ им. И.М. Сеченова» Минздрава России (Сеченовский Университет).

Время исследования

В исследование включены пациенты с АмИТ2, проходившие стационарное лечение с января 2006 по декабрь 2008 г.

## Изучаемые популяции

Использовался сплошной способ формирования изучаемой популяции. В исследование включены пациенты в возрасте 18–85 лет с манифестным тиреотоксикозом, отсутствием гиперваскуляризации при допплерографии, сниженным захватом технеция пертехнетата по данным сцинтиграфии щитовидной железы и низким уровнем АТ-рТТГ, приемом амиодарона не менее 1 мес или отменой его не более чем за 12 мес до развития АмИТ2. Средний срок приема препарата составил 104 [ 52; 135] дня.

Критериями исключения являлись: беременность и период лактации, выраженная почечная и печеночная недостаточность, психические расстройства.

## Дизайн исследования

Проведено ретроспективное исследование, включившее 38 пациентов с АмИТ2 (20 мужчин и 18 женщин). В зависимости от варианта медикаментозной терапии сформированы 3 группы пациентов: без терапии (n=19), получавшие ГК (n=11) или комбинацию ГК и тиреостатиков (n=8). Срок наблюдения составил 6–18 мес, включая период лечения. Прием ГК в стартовой дозе, эквивалентной преднизолону 30 [ 30; 40] мг/сут, продолжался до нормализации уровней свободных фракций тиреоидных гормонов, в дальнейшем осуществлялось снижение дозы на 5 мг каждые 7–10 дней до полной отмены. Продолжительность приема ГК составила 82 [ 71; 104] дня. Тиамазол назначался в дозе 30 [ 30; 40] мг/сут, длительность терапии составила 30 [ 24; 60] дней. У 7/8 человек прием тиамазола предшествовал назначению ГК.

## Описание медицинского вмешательства

Клинические методы обследования включали анализ анамнестических (суточная доза, длительность приема амиодарона, сопутствующая сердечно-сосудистая патология), антропометрических данных, оценку функционального состояния щитовидной железы: определение уровней тиреотропного гормона (ТТГ), свободных фракций тироксина (свТ4) и трийодтиронина (свТ3), АТ-ТПО, АТ-рТТГ. Всем пациентам выполнялись УЗИ щитовидной железы с допплерографией, сцинтиграфия с пертехнетатом технеция-99mTcO4. Медикаментозная терапия проводилась ГК или в комбинации с тиамазолом.

## Методы

Диагноз АмИТ2 устанавливался при наличии сниженного уровня ТТГ<0,4 мЕд/л, превышении значений свТ4>23,2 пмоль/л, референсном или свТ3>6,5 пмоль/л в сыворотке крови, отсутствии АТ-рТТГ (<1 МЕ/л), гиперваскуляризации при допплерографии, низком захвате пертехнетата технеция-99mTcO4 по данным сцинтиграфии щитовидной железы (<1%).

Уровни ТТГ (референс 0,4–4,0 мкМЕ/мл), свТ4 (референс 11,5–23,2 пмоль/л) определялись иммунохемилюминесцентным методом с помощью набора Immulite (США); свТ3 (референс 3,5–6,5 пмоль/л) — иммунохемилюминесцентным методом с помощью набора Вayer-ACS:180 (Германия); АТ-ТПО — иммуноферментным методом с помощью набора «Хема-Медика» (Россия), референс 0–60 МЕ/мл; АТ-рТТГ — радиорецепторным методом с помощью набора CIS Bio International (Франция), референс 0–1 МЕ/л. УЗИ щитовидной железы проводили аппаратом Hitachi EUB-405 plus с линейным датчиком 7,5 МГц, сцинтиграфию щитовидной железы с пертехнетатом технеция-99mTcO4 — с помощью вращающейся гамма-камеры GE 400T (General Electric, Бостон, Массачусетс, США). Эффективность терапии в группах оценивалась по времени достижения эутиреоза на фоне терапии и длительности тиреотоксикоза. В качестве дополнительного показателя оценивалась частота рецидива тиреотоксикоза после отмены ГК.

Ремиссией АмИТ2 являлось достижение эутиреоза — нормализация сывороточных уровней свТ4, свТ3 (подтверждалась дважды при снижении ГК и дважды после отмены с интервалом 1 мес) с последующим восстановлением референсного значения ТТГ>0,4 мЕд/л (обязательно через 1 и 3 мес после отмены ГК; в дальнейшем каждые 3 мес).

## Статистический анализ

Проверка количественных данных на нормальность распределения осуществлялась с помощью критерия Шапиро–Уилка и по величине асимметрии и эксцесса. Описательные статистики для количественных переменных представлены в виде медианы, интерквартильного диапазона (Mе [Q1; Q3]), для категориальных переменных — в процентах. Определение статистической значимости различий между группами для количественных данных проводилось с использованием критерия Краскела–Уоллиса с последующим применением роst-hос анализа — апостериорные парные сравнения с помощью критерия Манна–Уитни (критерия Данна) с применением поправки Бонферрони. Анализ номинальных данных производился с помощью точного критерия Фишера с последующим роst-hос анализом, сила связи между признаками определялась с помощью значения V Крамера. Для оценки изменений количественных показателей в связанных выборках использовался критерий Фридмана. Для оценки связи переменных использовался коэффициент ранговой корреляции Спирмена с интерпретацией по шкале Чеддока. Построение прогностических моделей производилось методом парной и множественной линейной регрессии, регрессии Кокса. Для оценки влияния факторов на выживаемость применялся метод Каплана–Мейера с использованием лог-ранк критерия Мантеля–Кокса. Различия считались статистически значимыми при значении p<0,05. Статистический анализ данных осуществлен при помощи пакета статистических программ SPSS v. 26 (SPSS, Chicago, IL, USA).

## Этическая экспертиза

Протокол исследования рассмотрен и одобрен на заседании комитета по этике при ассоциации медфармвузов при ПМГМУ им. И.М. Сеченова (протокол №10-08 от 11 декабря 2008 г.).

## РЕЗУЛЬТАТЫ

Проанализирована медицинская документация (истории болезни, амбулаторные карты) 38 пациентов с АмИТ2: 20 мужчин (52,6%) и 18 женщин (47,4%). До госпитализации в эндокринологический стационар все пациенты наблюдались кардиологом, 9/38 (23,7%) — эндокринологом. В зависимости от варианта терапии были сформированы 3 группы: без терапии (n=19), терапия ГК (n=11), комбинация ГК с тиамазолом (n=8).

Характеристика пациентов по группам представлена в таблице. В нашей выборке отмечалось равномерное распределение пациентов по полу. Средний возраст составил 62,0 [ 52,8; 66,3] года. В группе ГК ИМТ был достоверно меньшим по сравнению с группой без терапии (р=0,026). Большая часть пациентов (27/38 (71,1%)) были компенсированы по имеющейся сердечно-сосудистой патологии и не имели выраженной левожелудочковой недостаточности (медиана ФВЛЖ 62,0 [ 54,0; 66,0]%). У 26,3% (n=10) человек в анамнезе осуществлялось немедикаментозное восстановление сердечного ритма: электрическая кардиоверсия — 13,2% (n=5), радиочастотная абляция — 7,9% (n=3), имплантация искусственного водителя ритма — 5,3% (n=2). Виды нарушений ритма, послуживших причиной назначения амиодарона, представлены на рис. 1. У всех пациентов рецидивирование нарушений ритма сердца было перманентным проявлением АмИТ2. Негативных сердечно-сосудистых исходов за период тиреотоксикоза зарегистрировано не было. ФВЛЖ в динамике после разрешения АмИТ2 была известна у 6/38 пациентов (5 человек из группы без терапии и 1 из группы ГК), отмечалось статистически значимое ее увеличение (р=0,041).

**Table:** Таблица. Основные характеристики пациентов Примечание: НРС — нарушения ритма сердца; ФК — функциональный класс; ХСН — хроническая сердечная недостаточность; СД2 — сахарный диабет 2 типа; СКФ — скорость клубочковой фильтрации; Ам — амиодарон; свТ4манифест — уровень свТ4 при лабораторном подтверждении тиреотоксикоза; свТ3манифест — уровень свТ3 при лабораторном подтверждении тиреотоксикоза; свТ4/свТ3манифест — соотношение значений свТ4 к свТ3 при лабораторном подтверждении тиреотоксикоза; свТ4макс — максимальное регистрируемое значение свТ4; свТ3макс — максимальное регистрируемое значение свТ3; свТ4/свТ3макс — соотношение максимальных значений свТ4 к свТ3; свТ4ГК — уровень свТ4 при назначении глюкокортикоидов; свТ4ГК2нед — уровень свТ4 через 2 недели терапии глюкокортикоидами; свТ4ГК1мес — уровень свТ4 через 1 месяц терапии глюкокортикоидами; свТ4↓ГК — уровень свТ4 при снижении дозы глюкокортикоидов; ГКклиничАмИТ2 — время от возникновения клинической симптоматики тиреотоксикоза до назначения ГК; ГКлабАмИТ2 — время от лабораторного подтверждения тиреотоксикоза до назначения ГК; эутиреоз от начала терапии ГК — время достижения эутиреоза от начала терапии ГК; ИЗ99mTcO4 — индекс захвата пертехнетата технеция; АмИТ2клинич — длительность тиреотоксикоза от возникновения клинической симптоматики до нормализации уровней свТ4 и свТ3; АмИТ2лаб — длительность тиреотоксикоза от лабораторного подтверждения до нормализации уровней свТ4 и свТ3; клин-лаб — интервал между появлением клинических симптомов и лабораторным подтверждением тиреотоксикоза.*различия показателей между группами терапии статистически значимы (р<0,05).

Пол, n (%): мужчины	8 (42,11)	6 (54,55)	6 (75,00)	0,331
женщины	11 (57,89)	5 (45,45)	2 (25,00)
Возраст, лет, Mе [Q1; Q3]	62,0 [ 55,0; 66,5]	60,0 [ 55,0; 67,5]	60,0 [ 46,0; 65,5]	0,548
НРС, n (%): предсердные	17 (89,4)	7 (63,6)	8 (100)	0,320
желудочковые	1 (5,3)	2 (18,2)	
комбинированные	1 (5,3)	2 (18,2)	
Стенокардия II, III ФК, n (%)	8 (42,1)	6 (54,5)	5 (62,5)	0,587
ХСН II, III ФК NYHA, n (%)	6 (31,6)	3 (27,3)	3 (37,5)	0,894
ФВЛЖ, %, Mе [Q1; Q3]	62,0 [ 60,0–67,0]	59,0 [ 41,5–64,5]	61,5 [ 54,0–66,0]	0,621
Артериальная гипертензия, n (%)	14 (73,7)	10 (90,9)	5 (62,5)	0,331
Инфаркт миокарда, n (%)	1 (5,3)	4 (36,4)	1 (12,5)	0,076
Инсульт, n (%)	1 (5,3)	2 (18,2)	1 (12,5)	0,528
Миокардит, n (%)	2 (10,5)			0,348
Порок сердца, n (%)	3 (15,8)	2 (18,2)	1 (12,5)	0,945
ИМТ, кг/м², Mе [Q1; Q3]	31,00 [ 27,20; 33,00]	24,80 [ 24,08; 27,85]	28,25 [ 24,30; 30,97]	0,044*
СД2, n (%)	2 (10,53)		1 (12,50)	0,507
СКФCKD-EPI, мл/мин/1,73 м², Mе [Q1; Q3]	79,00 [ 68,50; 87,50]	65,00 [ 53,50; 79,50]	70,00 [ 66,00; 89,50]	0,205
Суточная доза Ам, мг, Mе [Q1; Q3]	200 [ 200; 200]	200 [ 200; 300]	300 [ 200; 400]	0,194
Длительность Ам, недели, Mе [Q1; Q3]	67,0 [ 13,5; 127,0]	64,0 [ 39,5; 128,0]	118,0 [ 104,0; 155,0]	0,181
Отмена Ам (до/при АмИТ2)	7 (36,8)/10 (52,6)	2 (18,2)/9 (81,8)	2 (25,0)/6 (75,0)	0,526
Время АмИТ2 от начала приема Ам, недели, Mе [Q1; Q3]	81,0 [ 50,0; 146,0]	119,0 [ 78,0; 144,0]	122,0 [ 105,0; 155,0]	0,325
Время АмИТ2 от отмены Ам, недели , Mе [Q1; Q3]	36,0 [ 30,0; 48,0] (n=7)	88,0 [ 83,0; 96,0] (n=3)	310,0 [ 10,0; 52,0] (n=2)	0,041*
ТТГ, мкМЕ/мл, Mе [Q1; Q3]	0,01 [ 0,10; 0,12]	0,01 [ 0,01; 0,02]	0,01 [ 0,01; 0,04]	0,076
свТ4манифест , пмоль/л, Mе [Q1; Q3]	28,3 [ 25,2; 32,5]	36,2 [ 33,0; 49,4]	53,1 [ 46,3; 68,5]	<0,001*
свТ3манифест , пмоль/л, Mе [Q1; Q3]	6,6 [ 6,3; 7,2] (n=10)	6,90 [ 5,6; 8,2] (n=2)	12,9 [ 8,2; 17,5] (n=2)	0,132
свТ4/свТ3манифест , Mе [Q1; Q3]	4,0 [ 3,0; 5,2]	5,1 [ 4,9; 5,2]	3,1 [ 22,6; 3,6]	0,198
свТ4макс, пмоль/л, Mе [Q1; Q3]	32,1 [ 28,0; 35,4]	38,4 [ 34,9; 52,6]	68,5[ 53,8; 104,2]	<0,001*
свТ3макс, пмоль/л, Mе [Q1; Q3]	7,0 [ 6,3; 7,2] (n=11)	8,2 [ 7,6; 8,5] (n=3)	34,6 [ 24,2; 40,4] (n=3)	0,020*
свТ4/свТ3макс, Mе [Q1; Q3]	3,96 [ 3,39; 5,26]	4,66 [ 4,40; 4,77]	2,90 [ 2,53–3,25]	0,110
свТ4ГК, пмоль/л, Mе [Q1; Q3]		38,1 [ 32,1; 58,4]	73,9 [ 42,2; 75,6]	0,032*
свТ4ГК2нед, пмоль/л, Mе [Q1; Q3]		28,7 [ 23,7; 37,0]	48,2 [ 33,3; 58,5]	0,039*
свТ4ГК1мес, пмоль/л, Mе [Q1; Q3]		23,4 [ 19,6; 29,3]	39,3 [ 22,4; 47,2]	0,099
свТ4↓ГК, пмоль/л, Mе [Q1; Q3]		23,4 [ 18,6; 24,1]	22,0 [ 19,5; 23,8]	0,745
АТ-ТПО, МЕ/мл, Mе [Q1; Q3]	24,5 [ 13,5; 89,3]	13,0 [ 12,0; 23,0]	16,6 [ 13,3; 19,8]	0,771
АТ-рТТГ, МЕ/Мл, Mе [Q1; Q3]	0,5 [ 0,4; 0,7] n=12	0,7 [ 0,6; 0,8] n=4	0,7 [ 0,4; 0,9] n=4	0,355
Объем ЩЖ, мл, Mе [Q1; Q3]	17,0 [ 15,0; 21,7]	16,3 [ 13,3; 17,0]	20,5 [ 15,0; 25,3]	0,331
ИЗ99mTcO4, %, Mе [Q1; Q3]	0,5 [ 0,3; 0,6]	0,4 [ 0,1; 0,6]	0,2 [ 0,1; 0,4]	0,550
ГКклиничАмИТ2, дни, Mе [Q1; Q3]		35,0 [ 26,5–52,0]	50,5 [ 32,5–75,0]	0,265
ГКлабАмИТ2, дни, Mе [Q1; Q3]		12,0 [ 3,5; 25,0]	20,0 [ 9,0; 48,0]	0,432
Доза ГК, мг/сут, Mе [Q1; Q3]		30 [ 25; 30]	30 [ 30; 40]	0,136
Эутиреоз от начала терапии ГК, дни, Mе [Q1; Q3]		27,0 [ 18,0; 34,5]	51,0 [ 22,5; 69,5]	0,047*
АмИТ2 клинич, дни, Mе [Q1; Q3]	57,0 [ 40,0; 103,5]	78,0 [ 46,0; 95,0]	102,5 [ 90,0; 122,5]	р2–3=0,020*
АмИТ2 лаб, дни, Mе [Q1; Q3]	30,0 [ 20,0; 57,0]	41,0 [ 26,0; 62,0]	75,0 [ 66,5; 107,5]	p2–3=0,016*
Клин-лаб, дни, Mе [Q1; Q3]	22,0 [ 14,0; 36,0]	24,0 [ 15,0; 32,5]	15,0 [ 14,0; 29,5]	0,881

**Figure:**
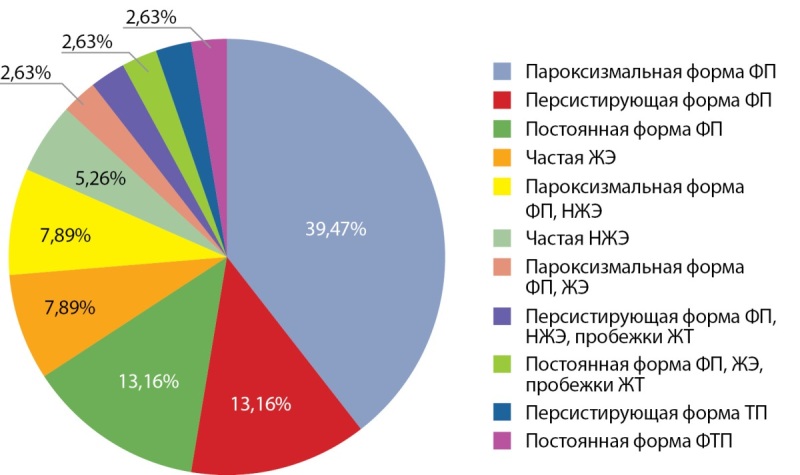
Рисунок 1. Виды нарушений ритма сердца при назначении амиодарона.Примечание: НЖЭ — наджелудочковая экстрасистолия, ФП — фибрилляция предсердий, ТП — трепетание предсердий, ФТП — фибрилляция и трепетание предсердий, ЖЭ — желудочковая экстрасистолия, ЖТ — желудочковая тахикардия.

Не было статистически значимых различий между группами по суточной дозе и длительности приема амиодарона (у 3 пациентов интервал составил 286, 386 и 1286 дней). В 28,9% (n=11) случаев терапия амиодароном была завершена до развития тиреотоксикоза, дальнейший прием препарата после подтверждения АмИТ2 сохранился только у 2 (5,3%) пациентов.

Тяжесть тиреотоксикоза условно оценивалась по максимальному уровню свТ4. В нашей выборке 60,5% (n=23) имели легкое течение АмИТ2 (свТ <40 пмоль/л), 31,6% (n=12) — умеренное (свТ4 40–80 пмоль/л), 7,9% (n=3) — тяжелое (свТ4>80 пмоль/л). Наиболее выраженным тиреотоксикоз оказался в группе комбинированного лечения: исходный и максимальный уровень свТ4, а также максимальный уровень свТ3 были значимо выше по сравнению с группой без терапии (р=0,001, р<0,001 и р=0,016 соответственно). С группой ГК-терапии статистически значимых различий по этим показателям не выявлено (р=0,547, р=0,190, р=0,402 соответственно). Уровень свТ4 при манифестации тиреотоксикоза в группе ГК был значимо выше, чем в группе без терапии, р=0,035.

Три группы не различались по объему щитовидной железы, индексу захвата пертехнетата ­технеция-99mTcO4, уровням АТ-ТПО, АТ-рТТГ.

В качестве ГК-терапии пациенты получали преднизолон, лишь в одном случае — метилпреднизолон в эквивалентной дозе. Не было различий между группами по стартовой дозе ГК, времени назначения ГК от появления клинической симптоматики (р=0,265) и лабораторного подтверждения тиреотоксикоза (р=0,432).

Уровень свТ4 перед назначением ГК был достоверно выше в группе комбинированной терапии, р=0,032. При оценке динамики уровня свТ4 на фоне терапии в группе ГК через 2 нед наблюдалось снижение значений с 38,1 [ 32,1; 58,4] до 28,7 [ 23,7; 37,0] пмоль/л (р=0,057), эутиреоз был достигнут у 36,4% (4/11) пациентов; достоверное снижение отмечено через 1 мес от начала терапии: с 38,1 [ 32,1; 58,4] до 23,4 [ 19,6; 29,3] пмоль/л (р=0,001), эутиреоз достигнут у 63,6% (7/11) пациентов. В группе комбинированной терапии через 2 нед от начала приема ГК снижение уровня свТ4 произошло с 73,9 [ 42,2; 75,6] до 48,2 [ 33,3; 58,5] пмоль/л (р=0,137), эутиреоз достигнут у 25% (2/8) пациентов; через 1 мес — с 73,9 [ 42,2; 75,6] до 39,3 [ 22,4; 47,2] пмоль/л (р<0,001), эутиреоз достигнут у 37,5% (3/8) пациентов (рис. 2).

**Figure:**
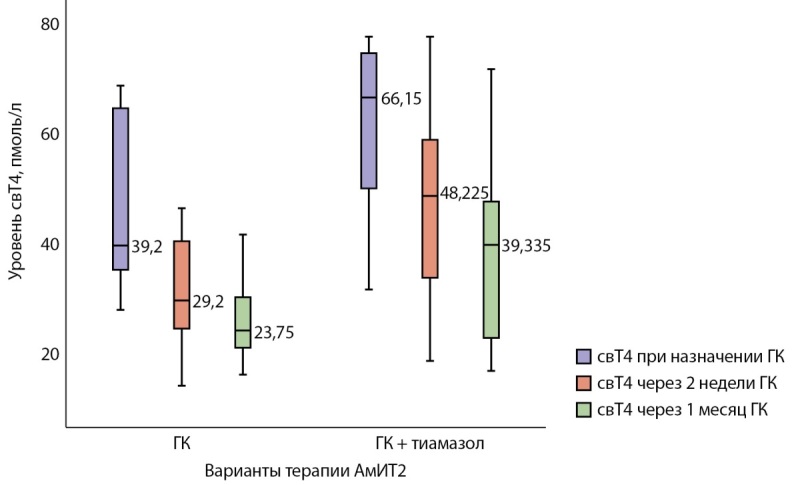
Рисунок 2. Динамика уровня свТ4 при назначении глюкокортикоидов.

Достоверных корреляционных связей между временем достижения эутиреоза в группе ГК с полом, ИМТ, возрастом, суточной дозой амиодарона, длительностью его приема, уровнем свТ4 при манифестации и назначении ГК, объемом щитовидной железы не установлено.

В группе комбинированного лечения при сравнении с группой ГК время достижения эутиреоза статистически значимо было большим (р=0,047), однако корреляционных связей с дозой (р=0,338) и длительностью приема тиамазола (р=0,911), уровнем свТ4 при манифестации (р=0,352) и назначении ГК (р=0,531), а также с полом, ИМТ, суточной дозой амиодарона и длительностью приема, объемом щитовидной железы не выявлено. В этой группе уровень свТ4 через 1 мес терапии ГК и время достижения эутиреоза коррелировали с возрастом: ρ=-0,857; p=0,007 и ρ=-0,786; p=0,021 соответственно. При оценке методом парной линейной регрессии подтверждена зависимость уровня свТ4 через 1 мес терапии ГК от возраста: увеличение возраста на 1 год предполагает уменьшение уровня свТ4 через 1 мес терапии ГК на 1,3 пмоль/л (константа=112,075, коэффициент регрессии В=-1,313; R²=0,627, р=0,019).

Длительность АмИТ2 от появления клинической симптоматики до нормализации свободных фракций тиреоидных гормонов в группе комбинированного лечения была большей по сравнению с группой ГК (р=0,020) (рис. 3). Выявленная корреляционная связь длительности тиреотоксикоза с объемом щитовидной железы (ρ=0,826; p=0,011) не была подтверждена методом парной линейной регрессии (R²=0,366, р=0,112). В группе ГК длительность тиреотоксикоза коррелировала со временем назначения ГК от начала клинической симптоматики (ρ=0,881; p<0,001); при линейном регрессионном анализе: константа=36,319, коэффициент регрессии В=0,920; R²=0,605, р=0,005. Статистически значимых различий по исходному уровню свТ4 и при назначении ГК в этой группе не было (р=0,109). С помощью лог-ранк критерия Мантеля–Кокса оценена зависимость достижения эутиреоза в течение 1 мес терапии ГК от времени назначения ГК (р=0,001): медиана срока общей выживаемости составила 102,0±31,5 (95% ДИ 40,2–163,8), средний срок достижения эутиреоза 83,8±12,1 (95% ДИ 60,1–107,6); при назначении ГК≤30 дней от начала клинической симптоматики тиреотоксикоза средний срок достижения эутиреоза составил 42,0±4,0 (95% ДИ 34,2–49,8) дня; при назначении ГК>30 дней от возникновения клинических симптомов — 107,7±11,1 (95% ДИ 85,9–129,6) дня (рис. 4).

**Figure:**
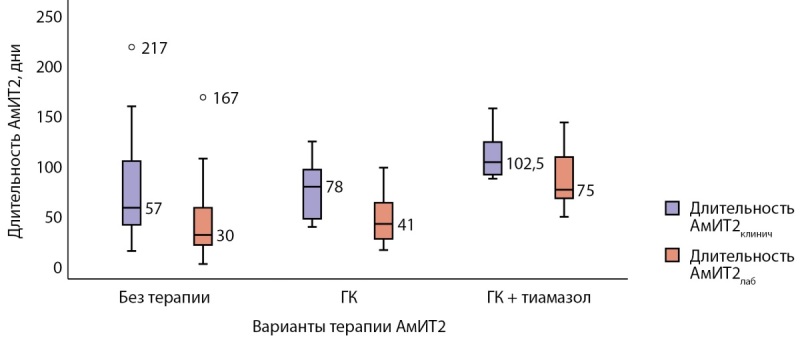
Рисунок 3. Длительность АмИТ2 при различных вариантах терапии.

**Figure:**
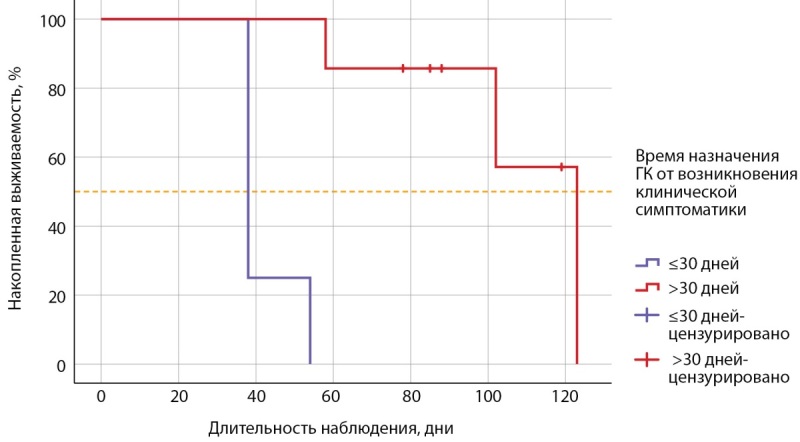
Рисунок 4. Кривая Каплана–Мейера, характеризующая выживаемость пациентов с недостижением эутиреоза в течение 1 месяца терапии глюкокортикоидами в зависимости от времени их назначения (группа ГК).

Длительность АмИТ2 от лабораторного подтверждения до достижения эутиреоза в группе без терапии коррелировала с уровнем свТ4 при манифестации тиреотоксикоза: ρ=0,587; p=0,008. При оценке зависимости показателей методом парной линейной регрессии отмечалась тенденция к достоверности: R²=0,203, р=0,053 (константа=-29,542, коэффициент регрессии В=2,505). При коррекции на интервал «клиническая симптоматика — лабораторное подтверждение» данная корреляция не была статистически значимой.

Значения выживаемости по длительности тиреотоксикоза в зависимости от вида терапии были сопоставлены с помощью кривых Каплана–Мейера. Медиана срока общей выживаемости составила 78,0±8,5 (95% ДИ 61,4–94,6), средний срок достижения эутиреоза — 82,2±6,9 (95% ДИ 68,6–95,9): в группе без терапии медиана составила 57,0±8,0 (95% ДИ 41,4–72,6), средний срок 75,4±11,6 (95% ДИ 52,6–98,2) дня; в группе ГК медиана составила 78,0±17,1 (95% ДИ 44,6–111,4) дня, средний срок достижения эутиреоза — 74,6±9,6 (95% ДИ 55,9–93,4) дня, в группе комбинированной терапии медиана составила 102,0±9,2 (95% ДИ 84,0–120,0) дня, средний срок достижения эутиреоза — 109±8,8 (95% ДИ: 91,8–126,2) дня ((Log Rank (Mantel–Cox)=0,372)) (рис. 5).

**Figure:**
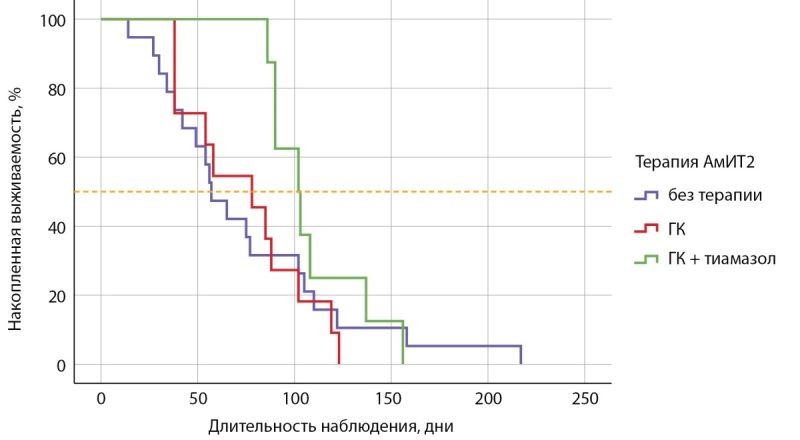
Рисунок 5. Кривая Каплана–Мейера, характеризующая выживаемость пациентов по длительности тиреотоксикоза в зависимости от вида терапии.

Изменение общей выживаемости пациентов по длительности тиреотоксикоза в зависимости от пола, возраста, ИМТ, исходного уровня свТ4, объема щитовидной железы, суточной дозы амиодарона, длительности его приема, наличия/отсутствия ГК-терапии, тиреостатической терапии оценено с помощью метода регрессии Кокса. В результате отбора предикторов методом исключения по Вальду статистической значимости ни для одного из факторов получено не было, переменные на последнем шаге: объем щитовидной железы (р=0,064), длительность приема амиодарона (р=0,097).

При дальнейшем наблюдении рецидивов АмИТ2 после отмены ГК не возникало. Транзиторный гипотиреоз отмечен у 4 пациентов (10,5%), с длительностью 46,00 [ 18,75; 226,25] нед и средним уровнем ТТГ 7,67 [ 6,07; 17,26] мкМЕ/мл. Через 12 мес после перенесенного АмИТ2 уровень ТТГ был известен у 68,4% (26/38) пациентов. Субклинический гипотиреоз наблюдался у 3 пациентов с максимальным значением ТТГ до 6,8 мкМЕ/мл, у остальных функция щитовидной железы оставалась сохранной, средний уровень ТТГ составил 3,36 [ 2,29; 4,00] мкМЕ/мл.

## ОБСУЖДЕНИЕ

Лечение АмИТ2, особенно в сочетании с сопутствующей ему кардиальной патологией, остается сложной задачей [9]. Обусловлено это как возможным тяжелым течением самого тиреотоксикоза, недостаточной и отсроченной восприимчивостью к медикаментозной терапии, так и выраженностью декомпенсации сопутствующей сердечно-сосудистой патологии, усиливающей негативные последствия АмИТ2 [23]. Возможно, быстрое достижение эутиреоза у таких пациентов имеет основное значение [24].

На сегодняшний день пероральные ГК — препараты выбора для лечения пациентов с умеренным и тяжелым течением АмИТ2 [9][10]. Они влияют на патогенетические механизмы развития АмИТ2, оказывают противовоспалительное, мембраностабилизирующее действия, подавляют цитотоксические и цитолитические реакции. Снижают конверсию Т4 в Т3, ингибируя активность 5’дейодиназы 1-го типа [25].

В 20% случаев АмИТ2 может разрешиться самопроизвольно [26]. Необходимость назначения ГК при субклинических и легких формах зависит от выраженности проявлений сердечно-сосудистой патологии и обсуждается совместно с кардиологом [27]. По результатам нашего исследования, у 17 пациентов группы без терапии максимальный уровень свТ4 не превышал 40 пмоль/л (легкое течение), лишь у двух — 50,2 и 90,8 пмоль/л; свТ3 (n=11) до 10,1 пмоль/л.

Выбор стартовой дозы ГК был обусловлен уровнем свТ4: большая — при умеренном и тяжелом течении АмИТ2. Средняя доза преднизолона 0,5 мг/кг (30–40 мг/сут), а также медиана времени достижения эутиреоза (32 [ 15,0; 45,5] дня) сопоставимы с результатами итальянских (28–40 дней) [20][22][24] и австралийских коллег (32–35 дней) [28]. На фоне терапии преднизолоном отмечалось статистически значимое снижение уровня свТ4 в обеих группах.

По мнению F. Bogazzi, N. Patel и соавт., высокий уровень свT4 связан с худшим ответом на медикаментозную терапию [22][28]. В 15–20% случаев АмИТ2 для достижения эутиреоза требуется более продолжительное время — до 6–8 мес [9][29]. В нашем исследовании различия во времени достижения эутиреоза в группе ГК и комбинированной терапии были статистически значимы, но достоверной корреляции с уровнем свТ4 не выявлено. Возможно, это связано с более поздним назначением ГК. Так, временной интервал от начала проявлений клинической симптоматики до назначения ГК в группе комбинированной терапии составил 50,5 [ 32,5–75,0] дня по сравнению с 35,0 [ 26,5–52,0] днями в группе ГК, значимость времени назначения ГК для терапевтического ответа подтвердилась с помощью лог-ранк критерия Мантеля–Кокса. Полученные медианы длительности тиреотоксикоза в этих группах 102,5 [ 90,0; 122,5] дня (минимум 86, максимум 156) и 78,0 [ 46,0; 95,0] дня (минимум 38, максимум 123) соответственно, сопоставимы с результатами F. Bogazzi, M. Isaacs, D. Cappellani и соавт.: 35–180 дней [22][30][31].

В ретроспективных исследованиях N. Patel, D. Conen и соавт. не выявлено различий по времени нормализации уровней свТ4 и ТТГ у пациентов в зависимости от того, получали они терапию преднизолоном или нет [28][32]. В связи с этим возникает вопрос, действительно ли преднизолон изменяет естественное течение заболевания, т. е. сокращает время достижения эутиреоза. В нашем исследовании при сравнении групп по длительности тиреотоксикоза на временном графике статистически значимых различий не получено, однако тяжесть заболевания была более выраженной в группе комбинированной терапии. Возможно, истинные максимальные значения свТ4 были известны не у всех пациентов группы без терапии. Наличие ГК-терапии не явилось предиктором и в нашей многофакторной прогностической модели. На наш взгляд, данный вопрос требует дальнейшего изучения. Интерес вызывает и информация о дозозависимом эффекте ГК при АмИТ2, применении высоких доз парентеральных форм ГК, подобных исследований крайне мало, проведены на малых выборках, полученные результаты противоречивы [33][34].

По данным проспективного исследования F. Bogazzi и соавт., уровень свT4 более 50 пг/мл и объем щитовидной железы более 12 мл значительно увеличивают время достижения эутиреоза, несмотря на проводимую ГК-терапию. Разрушение большего объема паренхимы приводит к более высоким значениям свТ4 и увеличивает длительность тиреотоксикоза [22]. Однако мы не можем предположить, какой процент этого объема будет вовлечен в деструктивный процесс в каждом конкретном случае. Превышение объема щитовидной железы в нашем исследовании отмечено у 1 пациента в группе ГК — 28 мл, 2 пациентов в группе комбинированного лечения — 25,3 и 29,9 мл и 3 в группе без терапии — 29,3, 64,8 и 84 мл. При этом он не коррелировал с максимальными значениями тиреоидных гормонов. В группе без терапии 2 пациентки со смешанным зобом 64,8 и 84 мл вызывали сомнения по поводу наличия смешанной формы АмИТ. Однако в обоих случаях отмечено преобладание повышения свТ4 (максимальные значения 37,4 и 36,5 пмоль/л соответственно) над свТ3 (до 7,0 пмоль/л), низкий уровень АТ-рТТГ, снижение эхогенности, отсутствие гиперваскуляризации паренхимы и узловых образований (с множественными полостными включениями), низкий захват 99mТс-пертехнетата по данным сцинтиграфии, что позволило классифицировать тиреотоксикоз в АмИТ2. Сцинтиграфия с 99mТс-технетрилом не проводилась. Прогностическим фактором ответа на ГК-терапию в нашем исследовании оказался возраст. Деструктивные формы тиреотоксикоза не сопровождаются увеличением синтеза тиреоидных гормонов и не требуют лечения тионамидами [24][28]. Обоснование добавления тиреостатиков заключается в том, что не всегда возможно точно дифференцировать тип АмИТ и, вероятно, наличием смешанных форм [9][10][17]. Тем не менее истинный АмИТ2 на сегодняшний день является наиболее распространенной формой тиреотоксикоза [12][13][35]. Ретроспективное исследование итальянских коллег продемонстрировало неэффективность применения тионамидов и отсроченное достижение эутиреоза [24]. В нашем исследовании отсутствие дифференциальной диагностики на амбулаторном этапе у пациентов в группе комбинированной терапии привело к нецелесообразному приему тиамазола, отсроченному назначению ГК и большей длительности тиреотоксикоза.

## Клиническая значимость результатов

Полученные результаты позволяют определить адекватную тактику ведения пациента и направление дальнейших исследований.

## Ограничения исследования

Ограничения исследования связаны с небольшим объемом выборки, ретроспективной оценкой имеющейся медицинской документации, неполным набором данных, отсутствием единого алгоритма динамики функции щитовидной железы, невозможностью оценки нежелательных событий, связанных с ГК-терапией, тиреоидного статуса и сердечно-сосудистых исходов в отдаленном периоде после перенесенного АмИТ2.

## Направления дальнейших исследований

Данный ретроспективный анализ был проведен как начальный этап для определения основных направлений и задач последующих проспективных исследований (оценка предикторов развития, длительности и тяжести АмИТ2, эффективности и безопасности различных стартовых доз преднизолона, факторов отсроченного ответа на терапию ГК, отдаленных сердечно-сосудистых исходов).

## ЗАКЛЮЧЕНИЕ

Оптимальным вариантом лечения при умеренном и тяжелом течении АмИТ2 является терапия ГК в средних дозах, при этом отсутствует четкая зависимость между временем достижения эутиреоидного состояния и длительностью приема, дозой преднизолона, объемом щитовидной железы. В качестве предиктора терапевтического ответа возможно рассматривать возраст пациента, важен временной интервал от возникновения клинической симптоматики до начала терапии ГК. Применение тиреостатических препаратов при АмИТ2 нецелесообразно. Создание прогностических моделей эффективности и длительности терапии обеспечит выбор оптимальной, безопасной тактики ведения пациентов и персонализированный подход.

## ДОПОЛНИТЕЛЬНАЯ ИНФОРМАЦИЯ

Источники финансирования. Работа выполнена по инициативе авторов без привлечения финансирования.

Конфликт интересов. Авторы декларируют отсутствие явных и потенциальных конфликтов интересов, связанных с содержанием настоящей статьи.

Участие авторов. Ермолаева А.С. — сбор и обработка материала, формирование электронной базы данных, статистическая обработка данных, анализ полученных результатов, написание основного текста статьи; Фадеев В.В. — научное руководство проводимого исследования, редактирование и финальное утверждение рукописи. Все авторы одобрили финальную версию статьи перед публикацией, выразили согласие нести ответственность за все аспекты работы, подразумевающую надлежащее изучение и решение вопросов, связанных с точностью или добросовестностью любой части работы.
